# Observations on Asphyxia Azotica

**Published:** 1802-06

**Authors:** 

**Affiliations:** Londonderry


### *Obſervations on Aſphyxia Azotica; communicated to Dr.* BRADLEY *by Dr.* PATTERSON *of Londonderry.*

THE 19th December, 1801, a little after eleven o'clock, a. m. I was called in a great hurry to ſee William M'Gertry and his wife, inhabitants of Derry; and was informed on the way, that the wife had been found dead about half an hour before fore on the floor of her bed-room, and the hufband nearly dead, leaning on the bed-fide. The woman certainly had been dead feveral hours, as the whole body was icy cold; no veftige of pulfe nor any refpiration remained, and every feature of the countenance teftified a complete extinction of vitality. She was about thirty years of age, and five months advanced in her fecond pregnancy.

The man, aged about thirty-three years, breathed obfcurely whilft fupported in a chair; his pulfe was not to be diftinguifhed; his countenance was pallid; his fkin cold; and he was unable to articulate a word. Some warm wine was given, fucceeded by ammonia; hot dry applications were made to the extremities; and fomentations were ufed to the abdomen. When he could fpeak, he complained of great pain about the epigaftric region, which feemed to be contracted, and the umbilical region tumefied. After taking fome warm tea, he vomited; and as he began to revive, he began to groan, and the pulfe became more perceptible. Warm broth and milk, which he took in turns, were the moft grateful articles of nutriment.

At twelve o'clock, p. m. he flumbered a little; his pulfe was ftill feeble and rather obfcure, with a coldnefs in the fkin. At nine o'clock, p. m. his pulfe was quite diftinct, his breathing fuller, and his fkin warmer. He moaned a good deal, and complained of ftomach ficknefs, naufea, and pain. He had a degree of thirft, to gratifiy which he prefers cold water mixed with milk.

Next day, his pulfe was from 100 to 104 in a minute, but equal; his fkin was warm; and he flumbered often during the preceding night. He vomited once in the night; complained yet of pain in the ftomach; had little or no appetite; was thirfty, but his head was free of any diforder. For nutriment he preferred milk gruel, which agreed with his ftomach; and he took a diflike to broth. He has had no evacuation from the inteftines, but he makes urine regularly. I ordered 5 gutt. tinct. opii. in aq. menth. every third hour, and to continue tranquil in bed. Thefe rules he obferved three days, when he was able to fit out of bed; and on the 25th, he could take food pretty well, but was not totally free from the pain about the region of the ftomach. This pain, however, gradually fubfided; and, in lefs than a fortnight, he was qualified to refume his occupation, which is that of a cloth-buyer for linendrapers.

The poor woman's fate is the more to be deplored, as fhe fell a victim to an act of conjugal affection in carrying a chaffing difh of peat coals to warm the bed-chamber for her hufband, who who had returned from market, after riding above thirty miles in cold frofty weather. The bed-chamber is a fmall room, about fix feet by eight, without a fire place, and with a clofe door and one window. Shortly after the coals were placed in the room the man went in, and he foon was followed by the woman, who was not long there until fhe called the fervant girl to bring her hufband a drink of water; and having taken the veffel with the water from the fervant, fhe locked the room door. No fooner was fhe undreffed than fhe fainted; and her hufband, who had juft laid down, fprung out of bed to her af-fiftance, and he alfo fainted; confequent to which, he had no farther recollection of what happened. Not long afterward, groans were heard by lodgers in an adjoining room, and by the fervant; but thefe groans being miftaken for thofe of a fick perfon in another part of the houfe, the place whence they really iffued was not examined, and the unfortunate woman confequently was fuffered to meet her mortal doom, whilft the wretched man was juft fnatched from the jaws of death, to witnefs and bewail the fad deftiny of his affectionate wife.

This recital feems to furnifh exercife for pathological inge-nuity, to develop the reafon why the female, who was but lit-tle younger than the male, and of an equally healthy good conftitution, fhould fo quickly come to a tragical end, whilft he retained the principles of life feveral hours after her's were totally abolifhed by the fame poifonous gas, which probably was carbonated hydrogen. Is the caufe to be fought for in the de-oxygenation of her blood, by the placental functions ab-ftracting oxygen for the fœtus in utero? Or, is the caufe to be derived from the excitability of the female brainular and nervous fyftem, whether fhe be in a pregnant or non-pregnant ftate, being eafier exhaufted by fudden impreffions than thofe of the male? Was being previoufly fo much in the frofty air, and the drink of cold water, any prefervatives to the man?

